# Enhancing Fullerene-Based Solar Cell Lifetimes by Addition of a Fullerene Dumbbell**

**DOI:** 10.1002/anie.201407310

**Published:** 2014-09-26

**Authors:** Bob C Schroeder, Zhe Li, Michael A Brady, Gregório Couto Faria, Raja Shahid Ashraf, Christopher J Takacs, John S Cowart, Duc T Duong, Kar Ho Chiu, Ching-Hong Tan, João T Cabral, Alberto Salleo, Michael L Chabinyc, James R Durrant, Iain McCulloch

**Affiliations:** Department of Chemistry and Centre for Plastic Electronics Imperial College London, London, SW7 2AZ (UK); Materials Department, University of California Santa Barbara Santa Barbara, California 93106 (USA); Department of Materials Science and Engineering, Stanford University 476 Lomita Mall, Stanford, California 94305 (USA); Department of Chemical Engineering and Centre for Plastic Electronics Imperial College London, London, SW7 2AZ (UK); São Carlos Physics Institute, University of São Paulo PO.Box: 369, 13560-970, São Carlos, SP (Brazil)

**Keywords:** fullerenes, lifetime, organic solar cells, photovoltaics, stability

## Abstract

Cost-effective, solution-processable organic photovoltaics (OPV) present an interesting alternative to inorganic silicon-based solar cells. However, one of the major remaining challenges of OPV devices is their lack of long-term operational stability, especially at elevated temperatures. The synthesis of a fullerene dumbbell and its use as an additive in the active layer of a PCDTBT:PCBM-based OPV device is reported. The addition of only 20 % of this novel fullerene not only leads to improved device efficiencies, but more importantly also to a dramatic increase in morphological stability under simulated operating conditions. Dynamic secondary ion mass spectrometry (DSIMS) and TEM are used, amongst other techniques, to elucidate the origins of the improved morphological stability.

The power conversion efficiency (PCE) of single-junction organic solar cells has increased significantly during the last decade to 9–10 %, now approaching the threshold considered necessary to commercialize the technology.^1^ During this period, the structural diversity of semiconducting donor polymers for solar cells has increased dramatically.^2^ These materials have helped enable an accelerated development of bulk heterojunction (BHJ) organic solar cells based on polymer donor materials and molecular fullerene derivatives. However, the development of electron-accepting materials that lead to BHJs with high PCE has been significantly slower.^3^ The most commonly used *n*-type acceptors to date remain [6,6]-phenyl-C_61_-butyric acid methyl ester (PC_61_BM) and its slightly larger counterpart PC_71_BM.

The morphology of polymer:PCBM BHJs is crucial for optimal PCE; it is important to achieve the right trade-off between domain size and finely interconnected network throughout the bulk of the active layer that allows efficient charge generation and extraction. The morphology can be controlled during device fabrication by employing solvent mixtures to deposit the active layer, adding high boiling solvent additives or alternatively by post-deposition treatments like solvent vapor or thermal annealing.^4^ However once the active layer is deposited and the desired morphology achieved, it is essential to “lock” that morphology in order to maintain high power efficiencies and to avoid device degradation over time. The “locking” of the active layer morphology is extremely non-trivial, due in part to the fullerene derivatives miscibility in the polymer phase, where they readily diffuse throughout the polymer on short timescales.^5^ Over an extended period of time or under thermal stress, the fullerene derivatives, especially PC_61_BM, form micron-scale crystallites within the polymer phase acting as charge traps and inhibiting efficient charge transport to the electrodes.^6^

One of the most common approaches to avoid aggregation of fullerenes into large domains is to chemically crosslink either or even both the materials in the BHJ after deposition.^7^ Crosslinking, usually triggered by external stimuli (e.g. heat or light), has been successfully used in lithography for decades, but has proven rather difficult to apply to organic semiconductor thin films. In most cases crosslinking the active layer inadvertently causes a significant performance decrease. We have recently demonstrated a new approach upon which the active layer morphology can be stabilized by the photoinduced [2+2] cycloaddition of the PC_61_BM derivatives, which results in covalent bonding between the fullerene groups.^8^ The strategy was found to hold generally for a variety of polymer–fullerene pairs.6c, ^9^ Light soaking significantly reduced the formation of PC_61_BM crystallites under thermal stress, and thus the device lifetime subsequently improved. Mechanistic studies indicated the enhancement in device thermal stability is primarily associated with inhibition of the nucleation of nanoscale PC_61_BM aggregates.6c These observations have motivated us to consider whether enhancement of device stability could also be achieved by the direct addition of covalently linked PC_61_BM dimers in solution prior to deposition of the active layer. Such an approach could provide a more structurally controlled and robust route to inhibit PC_61_BM aggregation, as well as enabling direct quantification of the degree of PC_61_BM dimerization required to enhance device stability, thus avoiding limitations associated with thermally induced dissociation observed for photoinduced PC_61_BM oligomers.6c

Our synthetic strategy was to make dumbbell-shaped dimeric fullerenes where the fullerenes are linked by an alkyl bridge between the ester functional group on PCBM. In a first step commercially available PCBM was hydrolyzed using a previously described literature procedure and the resulting PCBA was recovered as a black soot.^10^ The PCBA was immediately used in the next reaction step without any further purification. The use of thionyl chloride to convert the PCBA into the corresponding acyl chloride was disregarded because thionyl chloride potentially p-dopes carbon-based molecules like fullerenes, leading ultimately to poor device efficiencies.^11^ To prevent undesired doping with thionyl chloride, two equivalents of PCBA were reacted with one equivalent of ethylene glycol under mild reaction conditions using Steglich esterification. Isolating the desired (PCB)_2_C_2_ dumbbell in sufficient purity from the complex reaction mixture however proved difficult and gave unsatisfactory yields (<10 %). As a result of the impossibility to purify the poorly soluble PCBA starting material, it is likely that the acid and diol were not present in the required stoichiometric ratio, leading to the formation of a variety of compounds, difficult to separate. To circumvent these problems, the esterification reaction was performed in two steps as shown in Scheme 1. The crude PCBA was reacted with a large excess of diol (10 equivalents) to encourage the formation of PCBC_2_OH. The highly soluble PCBC_2_OH intermediate was readily purified by column chromatography and recovered in good yields. After a second esterification between PCBC_2_OH and PCBA, the (PCB)_2_C_2_ dumbbell could be successfully assembled and isolated in reasonable yield and excellent purity as evidenced by the ^1^H-MALDI and NMR spectra shown in Figures S5 and S10 in the Supporting Information. The singlet associated with the terminal methyl protons in PCBM appears at 3.67 ppm, whereas the singlet of the bridging methylene protons in (PCB)_2_C_2_ are shifted towards lower fields and appear at 4.26 ppm. All remaining signals associated with the aromatic and alkyl protons are identical in both cases and confirm that our dimerization approach has no effect on the chemical environment of the fullerenes.

**Scheme 1:**
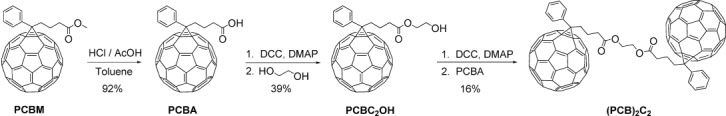
Synthesis of the PCBM dumbbell (PCB)_2_C_2_.

Because our goal was to blend the dimeric fullerenes with PCBM, we investigated the impact of the dimerization on their electronic structure. There have been numerous approaches to synthesize covalently bound fullerene dimers, however most of these synthetic procedures also modify the frontier energy levels of the fullerene dumbbells.^12^ From this perspective our controlled dimerization approach is advantageous, allowing the synthesis of structurally well-defined PCBM dumbbells with similar frontier energy levels to PCBM, as evidenced by density functional theory (DFT) calculations (Table S1) and cyclic voltammetry (CV) measurements (Figure S11). In both cases the HOMO was estimated at −5.94 eV and the LUMO at −3.85 eV, thus confirming the trend predicted by DFT calculations. The result might seem intuitive, but ensuring similar energy levels for both PCBM and the dimer (PCB)_2_C_2_ is crucial in order to achieve high PCE. In case the LUMO energy level of the dimer would be significantly lower than the one of PCBM, the added dimer would act as a charge trap, which would be detrimental for device performance.

To investigate the effect of addition of (PCB)_2_C_2_ on the active layer morphology and device performance, a blend of PCBM with the carbazole-based PCDTBT polymer was chosen as model system. PCDTBT is a high-performing amorphous polymer with a glass-transition temperature (*T*_g_≈106 °C) in neat films below the temperatures used here for examination of thermal stability.^13^ To determine the minimum weight percentage of (PCB)_2_C_2_ dumbbell necessary to stabilize the PCDTBT:PCBM blend under thermal stress, blends of PCDTBT:fullerene (1:2) were spin-coated on silicon substrates from chlorobenzene and thermally annealed at 140 °C for 1 hour. We have studied the effect of dimer content in the fullerene component, while maintaining the overall weight ratio of polymer:fullerene constant. From the optical micrographs presented in Figure S14, it is apparent that the addition of dimer leads to a more thermally stable blend. At low dimer loadings (<1 % by mass) the growth of micron-sized PCBM crystallites in the blend cannot be inhibited. However, the formation of micron-sized PCBM crystallites at temperatures above the *T*_g_ of the pure polymer is completely impeded upon addition of 20 % of (PCB)_2_C_2_ (Figure 1), and indeed the number density of the PCBM crystallites is suppressed by over 50 % by adding only 5 % of dimer to the blend (Figure 2 A). This low threshold is consistent with our previous conclusion that the primary role of the PC_61_BM oligomers, respectively (PCB)_2_C_2_, is to frustrate and eventually inhibit PC_61_BM crystal nucleation, with such nucleation processes often being hindered by low compositions of additive materials.6c, ^14^

**Figure 1:**
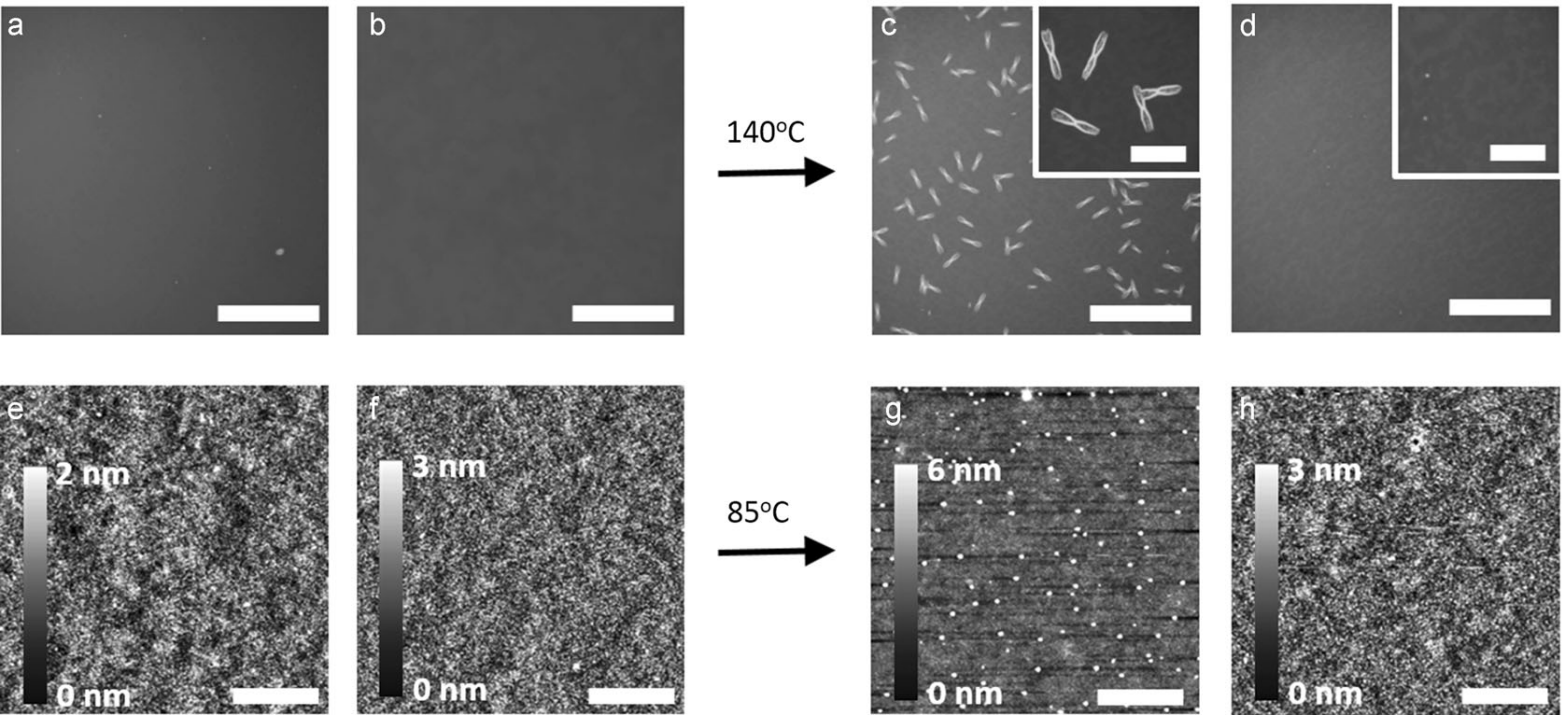
Optical images of a) PCDTBT:PCBM as-cast blend films (scale bar=200 μm); b) PCDTBT:PCBM:(PCB)_2_C_2_ (20 %) as-cast films (scale bar=200 μm); c) PCDTBT:PCBM blend films after thermal annealing at 140 °C for 1 h (scale bar=200 μm)and d) PCDTBT:PCBM:(PCB)_2_C_2_ (20 %) blend films after thermal annealing at 140 °C for 1 h on SiO_x_ substrates (scale bar=200 μm). The insets in (c,d) highlight the drastic differences in PCBM crystal formation in the annealed blend films with and without the (PCB)_2_C_2_ (scale bar=40 μm). AFM images of e) PCDTBT:PCBM as-cast blend films (scale bar=5.0 μm); f) PCDTBT:PCBM:(PCB)_2_C_2_ (20 %) as-cast films (scale bar=5.0 μm); g) PCDTBT:PCBM blend films after thermal annealing at 85 °C for 1 h (scale bar=5.0 μm); and h) PCDTBT:PCBM:(PCB)_2_C_2_ (20 %) blend films after thermal annealing at 85 °C for 1 h on PEDOT:PSS substrates (scale bar=5.0 μm).

**Figure 2:**
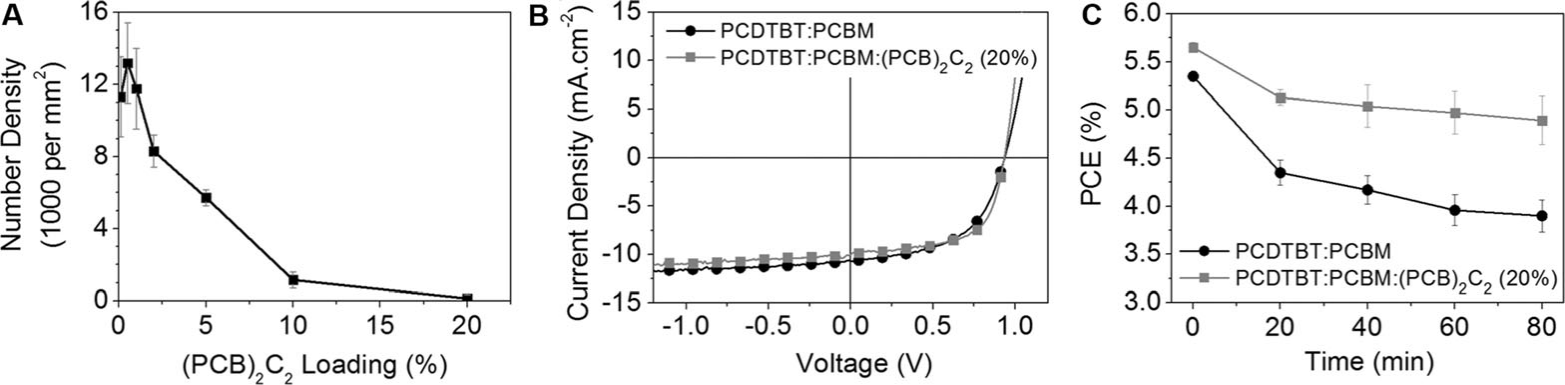
A) Number density of micron-sized PCBM crystallites formed in blend films after thermal annealing at 140 °C for 1 h on SiO_*x*_/Si substrates, plotted as a function of the (PCB)_2_C_2_ dimer loading. B) Comparison of the *J*–*V* characteristics of optimized conventional PCDTBT:PCBM devices and PCDTBT:PCBM:(PCB)_2_C_2_ (20 %). C) Degradation of solar cell PCE as a function of annealing time at 85 °C in nitrogen atmosphere. The error bars represent the spread in degradation kinetics of three typical devices

The effect of (PCB)_2_C_2_ on the nanomorphological behavior upon thermal stress analogous to solar-cell-operating conditions was also investigated. Our previous studies have shown that thermal stress at temperatures relevant to device operation does not lead to micron-size crystallites, but instead results in the formation of nanometer-sized PCBM features in PCDTBT:PCBM blend films.6c The evolution of the blend morphology upon thermal annealing at 85 °C, customary for standard thermal stress tests, on PEDOT:PSS substrates is shown in Figure 1.^15^ As revealed by AFM, as cast samples are relatively smooth and featureless, with a similar surface roughness for both with and without (PCB)_2_C_2_. Upon thermal annealing at 85 °C for 1 h, the samples without (PCB)_2_C_2_ exhibit densely formed PCBM crystallites of the order of 150 nm in diameter and 30 nm in height. We have previously assigned these nanoscale features to PCBM aggregates, and correlated their appearance with the degradation of device efficiency under modest thermal stress.6c In clear contrast, the samples with 20 % (PCB)_2_C_2_ show drastically suppressed PCBM aggregation with no measurable change in film surface morphology after thermal treatment.

The film morphology was further probed by bright-field transmission microscopy (TEM). At 80 °C, both blend films, with and without (PCB)_2_C_2_, present very comparable features. The non-(PCB)_2_C_2_ containing blend contains spherical aggregates dispersed homogenously throughout the film (Figures S15 to S17), with diameters ranging from about 150–250 nm. The (PCB)_2_C_2_ containing blend showed comparable features, but a much lower density of fullerene nuclei of approximate diameter of 100–150 nm was found. This modest difference suggests that the observed spherical aggregates in the non-(PCB)_2_C_2_ containing blend might play a key role in PCBM crystal nucleation and device degradation even though the exact mechanism is not yet elucidated. Upon thermal annealing at 140 °C, the formation of fullerene nuclei varying in size (about 50–100, 100–150, and 150–200 nm) was observed in the non-(PCB)_2_C_2_ containing blend. More importantly however, we observed the formation of needle-like structures, similar to the ones previously observed in optical microscopy image, but much smaller in size (50–100 nm in length and 10 nm in width). In the annealed blend film containing 20 % of (PCB)_2_C_2_ these sheaf-like structures were not observed, but the morphology was instead characterized by the presence of spherical aggregates, on average smaller than the ones observed prior to 140 °C annealing. These results are in further agreement with our previously made observation that a low concentration of (PCB)_2_C_2_ in the fullerene phase is stabilizing the blend nanomorphology and seems to influence the shape and number density of PCBM aggregates under modest thermal stress.

Knowing that the PCBM dumbbell has a positive effect on the thermal stability of active layer blend films, we focused on its effect on the performance of PCDTBT:PCBM solar cells. A standardized thermal stress of 85 °C was applied to the devices under nitrogen atmosphere, with the current–voltage (*J*–*V*) curve recorded using repeated dark thermal annealing/room-temperature device testing cycles. For *J*–*V* measurements, devices were exposed under a solar simulator for less than 30 seconds during each measurement to minimize the effect of PCBM photo-oligomerization. Figure 2 demonstrates the impact of a low percentage of PCBM dimers on the efficiency and thermal stability of PCDTBT:PCBM devices. Devices containing 20 % of (PCB)_2_C_2_ show a modest improvement in efficiency compared to devices without dimer. This improvement is primarily due to an enhancement in fill factor (FF) from around 0.56 to 0.62, whereas both *V*_oc_ and *J*_sc_ are nearly identical in both sets of devices (Table S2). The cause of improved FF is not obvious from the AFM images (e) and (f) in Figure 1, but is likely due to an improvement in the blend morphology induced by the dimers during film deposition. For the devices fabricated without (PCB)_2_C_2_, a rapid degradation in device performance was observed upon thermal stress, with a 20 % loss in power conversion efficiency (PCE) within the first 25 min, primarily due a loss of fill factor, and likely linked to the densely formed nanoscale PCBM aggregates. Remarkably, devices containing 20 % of the (PCB)_2_C_2_ show significantly improved thermal device stability with a reduction in the PCE by 20 % occurring only after 2000 minutes. It is thus apparent that the device thermal stability of conventional PCDTBT:PCBM devices can be enhanced by an order of magnitude by using a low percentage of (PCB)_2_C_2_, at least under the thermal stress conditions and time scale studied herein.

Given that the addition of (PCB)_2_C_2_ not only enhances the long-term stability, but also the device efficiency, we evaluated the potential to further enhance the performance by systematically increasing the PCBM dimer loading from 0 % (100 % of PCBM) to 100 % (0 % of PCBM) while keeping the total concentration of fullerene in the active layer blend constant. The initial device *J*–*V* characteristics, as a function of the (PCB)_2_C_2_ loading, are shown in Figure S18. From 0 to 20 % of (PCB)_2_C_2_ loading, modest increases in PCE are observed because of enhanced FF values. However, further increase in dimer loading results in decreasing device PCE, caused by decreases in both *J*_sc_ and FF (see Table S2 in the Supporting Information for detailed device parameters). The loss in PCE results in part from a higher series resistance, indicative of a drop in electron mobility in the fullerene phase with increasing PCBM dimer loading. To confirm this assumption, organic field-effect transistors (OFET) were built to measure the electron mobility as a function of (PCB)_2_C_2_ loading. The electron mobility of a neat PCBM film was found to be around 1×10^−3^ cm^2^ V^−1^ s^−1^, which is in good agreement with previously reported literature values.^16^ As evidenced from Figure 3 A, a sharp drop in electron mobility is observed at (PCB)_2_C_2_ loadings exceeding 10–20 %, which correlates well with the lower *J*_sc_ measured at higher dumbbell loadings and the resulting drop in PCE. These findings are in line with previous work by Distler et al., who demonstrated that photodimerization of PCBM causes a significant reduction of charge carrier mobility in a polymer–fullerene system, resulting in a reversible degradation of device efficiency under constant illumination.^17^ The electron mobility measurements in conjunction with the device stability data therefore conclusively show that there is an optimal (PCB)_2_C_2_ loading around 20 % at which both the device efficiency and the device lifetime can be simultaneously enhanced.

**Figure 3:**
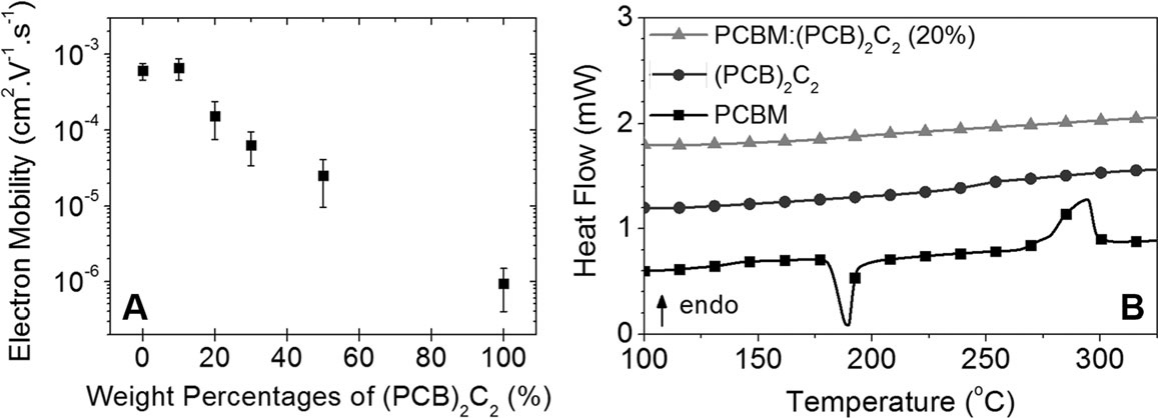
A) Electron mobility of PCBM:(PCB)_2_C_2_ measured in organic field-effect transistors as a function of (PCB)_2_C_2_ loading. B) DSC first heating thermogram of PCBM, (PCB)_2_C_2_, and PCBM:(PCB)_2_C_2_ (20 %) after the samples were thermally annealed at 85 °C during two hours. Heating rate 10 °C min^−1^.

Based on the aforementioned results, the addition of (PCB)_2_C_2_ leads to improved power conversion efficiencies, mainly because the growth of nanometer-sized PCBM crystallites in the active layer under thermal stress is prevented as evidenced by AFM measurements. To further investigate by what mechanism this crystallite growth is suppressed, we have carried out interdiffusion measurements. We hypothesized above that the addition of (PCB)_2_C_2_ would lead to lower mass diffusion coefficient, thus slowing down and eventually preventing, the growth of PCBM nanocrystallites. The diffusion of (PCB)_2_C_2_ from a bulk heterojunction blend with PCDTBT into a neat PCDTBT thin film was thus investigated with dynamic secondary ion mass spectrometry as a function of annealing temperature (Figure 4). The bilayers remained stable in composition, as prepared from solution casting and film lamination, until the annealing temperature approached the *T*_g_ of PCDTBT (beginning at 100 °C; Figure S19). At temperatures near or slightly below *T*_g_, the (PCB)_2_C_2_ infiltrated only partially into the originally neat PCDTBT film, with a decreasing dimer concentration with diffusion distance. At temperatures above *T*_g_, the (PCB)_2_C_2_ diffused entirely across the PCDTBT film with uniform concentration. These results suggest that the molecular mobility of PCDTBT is critical in determining the ability for the dimer molecule to traverse the polymer phase. While (PCB)_2_C_2_ and PCBM show qualitatively the same behavior with PCDTBT, we cannot rule out that the diffusion of the former is slowed down, as the DSIMS measurement provides only a bound for the diffusion constant. We can state however that it is not slowed so much as to retard diffusion at timescales commonly used in processing of BHJs, that is, minutes. More critically, the miscibility in PCDTBT seems unaffected by the dimerization process. These results imply that in blends with PCDTBT, dimerized and undimerized PCBM show similar diffusion kinetics and suggest that rather than slowing growth of aggregates and crystals by mass diffusion in such blends, that the stability of the microstructure is achieved through frustration of molecular packing of PCBM by (PCB)_2_C_2_. Grazing incidence wide-angle X-ray scattering (GIWAXS) measurements performed on PCDTBT:fullerene blends revealed only some weak ordering from PCDTBT, with (100) and (200) peaks that are broad and weakly observable in the out-of-plane and in-plane directions (Figure S25). The large, broad aggregate peak centered at 1.4 Å^−1^ has contributions from the weakly ordered/aggregated PCBM and possibly (PCB)_2_C_2_, as well as from the amorphous regions of PCDTBT. No diffraction peaks related to the spherical fullerene aggregates observed in TEM images could be identified, which is likely because these are only very weakly ordered. The hypothesis of frustrated molecular packing is further supported by differential scanning calorimetric (DSC) measurements as evidenced from Figures S20–S24. Pure PCBM exhibits a sharp melting peak at 280 °C with an associated enthalpy of fusion of 14.8 J g^−1^. However upon addition of 20 % of (PCB)_2_C_2_ dumbbell, it is possible to quench the PCBM:(PCB)_2_C_2_ blend in a glassy state (*T*_g_ of about 200 °C) and even after 2 h of thermal annealing at 85 °C the sample did not return to a crystalline state comparable to the neat PCBM sample (Figure 3 B).

**Figure 4:**
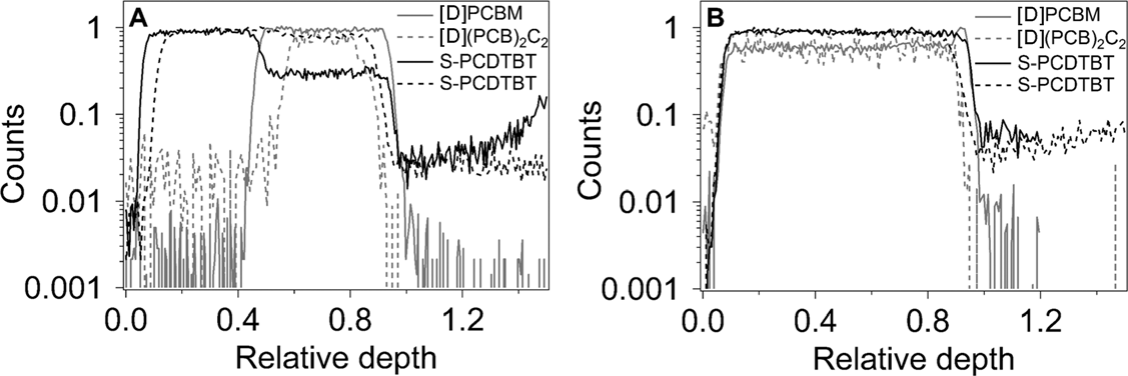
Dynamic secondary ion mass spectrometry profiles of ^2^H and ^32^S in a PCDTBT:[D]PCBM bilayer (solid lines) and in a PCDTBT:(BHJ PCDTBT:[D](PCB)_2_C_2_) bilayer (dotted line) fabricated on a SiO_2_/Si substrate. A) Sharp changes in the ^2^H and ^32^S counts are observed in the as-cast bilayer structures. B) The homogenous distributions of the deuterated species is achieved at an annealing temperature (140 °C) exceeding the *T*_g_ of the polymers.

These results demonstrate conclusively that the addition of a low-weight percentage of modified fullerene to a polymer–fullerene active layer does not negatively affect the PCE of BHJs and can lead to the significantly enhanced morphological stability. The underlying mechanism appears to be, not a significant reduction in the mass diffusion of the fullerene, but rather the frustration of fullerene nanocrystal nucleation. We find that there is an optimal loading, of approximately 20 % fullerene dimer, that yields a device lifetime increase of around 20 % under realistic operating conditions. In contrast with our previously reported strategy of photo-induced fullerene oligomerisation in the active layer, which yields a time-dependent fullerene–oligomer population (as a function of time and illumination), this approach is robust to environmental conditions and thus potentially more attractive for practical implementation.6c, 8

## Supporting Information

Supporting information for this article, including synthetic details and additional graphs (CV, UV/Vis, OPV, DSC, OFET) and images (microscopy, TEM), is available on the WWW under http://dx.doi. org/10.1002/anie.201407310.

miscellaneous_information
