# Supplementary material for: Enhancing Fullerene-Based Solar Cell Lifetimes by Addition of a Fullerene Dumbbell
Source: Angew Chem Int Ed Engl. 2014 Sep 26;53(47):12870–5. doi: 10.1002/anie.201407310 (PMC4241035; doi:10.1002/anie.201407310)
Supplement: Supplementary file 1 — miscellaneous_information [file anie0053-12870-SD1.pdf]

Angewandte Chemie
Eine Zeitschrift der Gesellschaft Deutscher Chemiker

Supporting Information
© Wiley-VCH 2014
69451 Weinheim, Germany

Enhancing Fullerene-Based Solar Cell Lifetimes by Addition of a Fullerene Dumbbell**

Bob C. Schroeder, Zhe Li,* Michael A. Brady, Gregório Couto Faria, Raja Shahid Ashraf, Christopher J. Takacs, John S. Cowart, Duc T. Duong, Kar Ho Chiu, Ching-Hong Tan, João T. Cabral, Alberto Salleo, Michael L. Chabinyc, James R. Durrant, and Iain McCulloch*

anie_201407310_sm_miscellaneous_information.pdf

Experimental details 2

Synthetic details 3

Cyclic voltammetry, UV-vis. absorption and computational data 9

Microscopy and TEM images 11

OPV data 14

DSIMS profile and DSC traces 15

GIXD data 18

OFET transfer curves 19

PCBM was purchased from Solenne BV, all other chemicals and solvents were purchased from Sigma-Aldrich and used as received. PCBA was synthesized according to a literature procedure.[1] ^{1}H NMR and ^{13}C NMR spectra were recorded on a Bruker 400 spectrometer at 298 K and chemical shifts are given in ppm. UV-*vis.* absorption spectra were recorded on a UV-1800 Shimadzu UV-*vis.* spectrometer. Cyclic voltammograms were obtained using an Autolab PGSTAT101 potentiostat with a standard three-electrode setup with a platinum disk working electrode and a Ag/Ag^+ reference electrode calibrated against Fc/Fc^+. The measurements were carried out with approximately 0.5 $\times 10^{-3}$ M solutions of PCBM, respectively $(PCB)_2C_2$, in a mixture of anhydrous and deoxygenated acetonitrile:*o*-dichlorobenzene (1:5 v/v) with 0.1 M tetrabutylammonium hexafluorophosphate as the supporting electrolyte at a scan rate of 100 mV/s. HOMO and LUMO energy values were obtained using equations:

$$E_{LUMO} = -(E_{red} - E_{Fc} + 4.8)\ \text{eV and } E_{HOMO} = -(E_{ox} - E_{Fc} + 4.8)\ \text{eV}$$

PCDBT was supplied by 1-Materials Inc. ($M_w \approx 21.6$ kg.mol^{-1}; $D_w \approx 5.5$) and PCBM was purchased from Nano-C Inc. PCDTBT and PCBM, respectively $(PCB)_2C_2$, (1:2 weight ratio) were co-dissolved in chlorobenzene and stirred for 24 hours at 55°C inside a nitrogen filled glovebox. The total solution concentration was 25 mg.mL^{-1}. All devices were prepared with a conventional device architecture (ITO/PEDOT:PSS/PCDTBT:PCBM/Ca/Al), the active layer was deposited via spincoating at room temperature and the film thickness was measured to be around 80 nm.

Matrix-assisted laser desorption ionization (MALDI) mass spectra (MS) were obtained using a Micromass MALDI mass spectrometer equipped with a time-of-flight (ToF) analyzer. All mass spectra were acquired in negative mode. *trans*-2-[3-(4-*tert*-Butylphenyl)-2-methyl-2-propenylidene]malononitrile was used matrix. The sample was deposited together with the matrix and C_{60} as internal reference from *o*-dichlorobenzene solution by drop-casting directly on the MALDI plate. mode.

DSC experiments were carried out with a TA Instruments DSC Q20. The samples for the thermal annealing DSC measurements, were prepared by loading the DSC pan with the analyte and heating the sample multiple times to 325°C and until consecutive heating cycles were identical. The sample was then annealed at 85°C during two hours in the DSC machine and heated to 325°C to induce the melt. Atomic force miscroscopy (AFM) was performed using an Asylum Research MFP 3D AFM, using NanoWorld Pointprobe Al-coated, non-contacted mode Si cantilevers, with a resonance frequency of 190 kHz and a spring constant of 48 N/m.

Dynamic secondary ion mass spectrometry (SIMS) was performed using a Physical Electronics 6650 quadropole instrument. Substrates were cooled on a cryostage (liquid nitrogen) for ~30 min prior to measurement. A 2kV O_2^+ beam at ~40-50 nA was rastered across a 300 µm x 300 µm area, of which only the middle 15% of the surface area was analyzed for composition through collection of negative secondary ions.

2D GIWAXS was conducted at beamline 7.3.3 at the Advanced Light Source (Lawrence Berkeley National Laboratory), using a Pilatus 1M CCD detector at grazing incidence with a photon energy of 10 keV. Samples were kept under helium atmosphere to minimize sample radiation damage and the low-q background in the collected images. Films were typically exposed for 60-90 s at incident angles of 0.10-0.18°.

Thin films transistors were fabricated by spin casting PCBM:$(PCB)_2C_2$ solutions onto solvent cleaned silicon substrates with a 200 nm layer of thermally grown oxide and patterned gold contacts with channel lengths between 20 to 30 µm. Transfer curves were collected under vacuum (≈10^{-3} Torr) and electron mobilities were extracted in the saturation regime ($V_D > 60$ V).

[6,6]-Phenyl-C61-butyric acid 2-hydroxyethyl ester ($PCBC_2OH$). [6,6]-Phenyl-C61-butyric acid (**PCBA**) (600 mg, 0.67 mmol) was added to 100 mL of anhydrous *o*-dichlorobenzene and sonicated during 20 minutes. After sonication the brownish suspension was heated to 110°C for two hours in order to fully dissolve the PCBA. The resulting black solution was cooled to 0°C in an ice-bath before ethylene glycol (1 mL, 17.88 mmol) and 4-Dimethylaminopyridine (8.2 mg, 0.07 mmol) were added. At last *N,N'*-Dicyclohexylcarbodiimide (152 mg, 0.74 mmol) was added to the reaction mixture, which was allowed to warm to room temperature and stirred overnight. The solvent was removed from the reaction mixture by rotary evaporation. The recovered crude product was purified by column chromatography on silica gel using a mixture of toluene:pyridine (95:5) as mobile phase. The recovered fractions were concentrated and precipitated into cold methanol. The title compound was recovered as a brown solid after filtration (247 mg, 0.26 mmol, 39%). ^{1}H NMR (400 MHz, chloroform-*d*) δ ppm 7.97 - 7.91 (m, 2 H) 7.60 - 7.46 (m, 3 H) 4.27 - 4.21 (m, 2 H) 3.88 - 3.77 (m, 2 H) 3.01 – 2.87 (m, 2 H) 2.59 (t, *J*=7.3 Hz, 2 H) 2.29 - 2.15 (m, 2 H) 1.80 (br s, 1 H). MS (MALDI-ToF): *m/z* calcd for $C_{73}H_{16}O_3$ (M^{-}) 940.1, 941.1, 942.1, 943.1 found 940.1, 941.1, 942.1, 943.1.

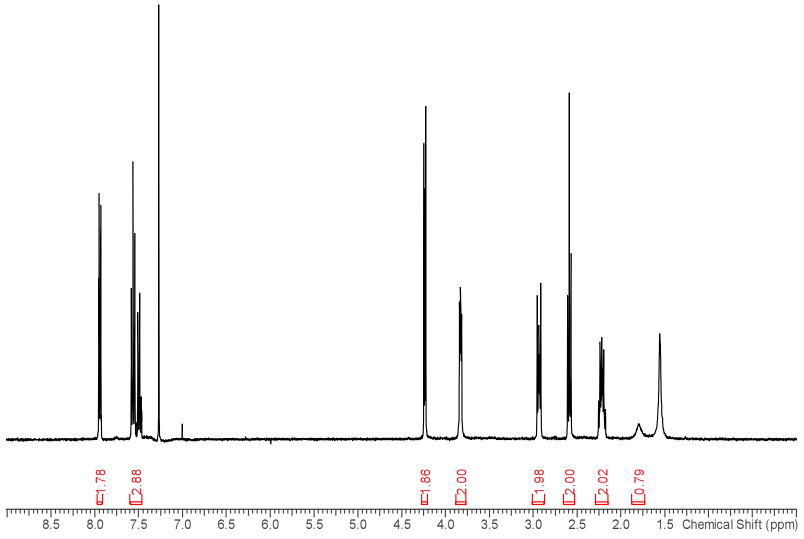

Figure S1. ^{1}H-NMR spectra of **$PCBC_2OH$** recorded at 293 K in chloroform-*d*.

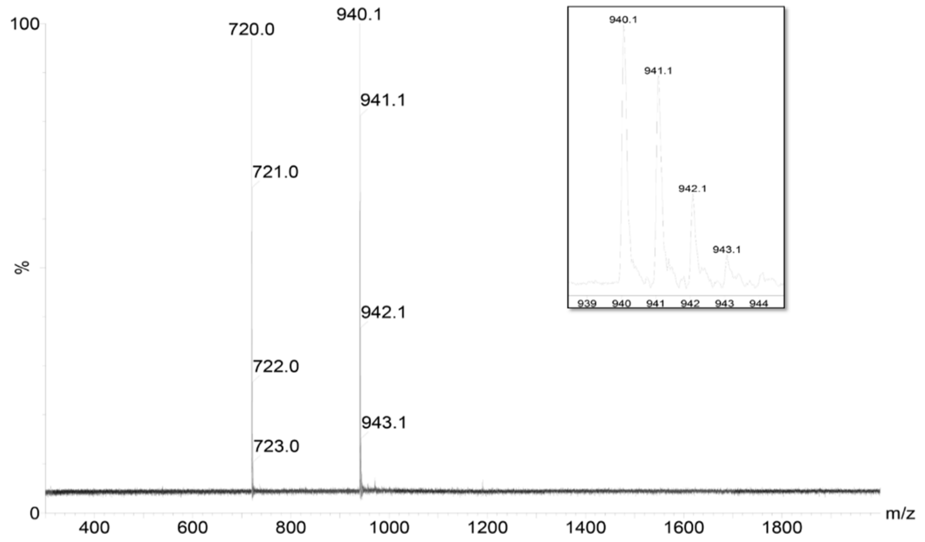

Figure S2. Negative ion MALDI mass spectrum of **$PCBC_2OH$**, using C_{60} (m/z: 720.0) as internal calibrant. The inset shows a zoomed view of the molecular ion peak.

Bis-[6,6]-phenyl-C61-butyric acid ethane-1,2-diyl ester ($(PCB)_2C_2$).

$PCBC_2OH$ (190 mg, 0.20 mmol) was dissolved in 100 mL of anhydrous *o*-dichlorobenze and cooled to 0°C. PCBA (362 mg, 0.40 mmol) and 4-Dimethylaminopyridine (4.9 mg, 0.04 mmol) were added, before *N,N'*-Dicyclohexylcarbodiimide (92 mg, 0.44 mmol) was added. The reaction mixture was allowed to warm to room temperature and stirred overnight. Afterwards the solvent was removed from the reaction mixture and the recovered crude product was purified by column chromatography on silica gel using toluene as eluent. The recovered fractions were concentrated and precipitated into methanol. The title compound was recovered as a dark brown solid after filtration (58 mg, 0.03 mmol, 16%). ^1H NMR (400 MHz, 1,1,2,2-tetrachloroethane-d_2) δ ppm 7.91 - 7. 86 (m, 2 H) 7.55 - 7.42 (m, 3 H) 4.22 (s, 2 H) 2.91 – 2.80 (m, 2 H) 2.51 (t, *J*=7.3 Hz, 2 H) 2.18 -1.94 (m, 2 H). ^{13}C NMR (100 MHz, TCE-d_2) δ ppm 207.5, 173.2, 149.0, 148.0, 146.1, 145.4, 145.4, 145.3, 145.2, 145.0, 144.9, 144.7, 144.6, 144.2, 144.0, 143.2, 143.2, 143.1, 142.4, 142.4, 142.3, 141.2, 140.9, 138.2, 137.8, 136.9, 136.5, 132.3, 128.8, 128.5, 123.8, 120.6, 80.1, 52.1, 34.1, 33.8, 22.5. MS (MALDI-ToF): *m/z* calcd for $C_{144}H_{26}O_4$ ($M^{\cdot -}$) 1819.2, 1820.2, 1818.2, 1821.2 found 1818.7, 1819.6, 1817.7, 1820.6.

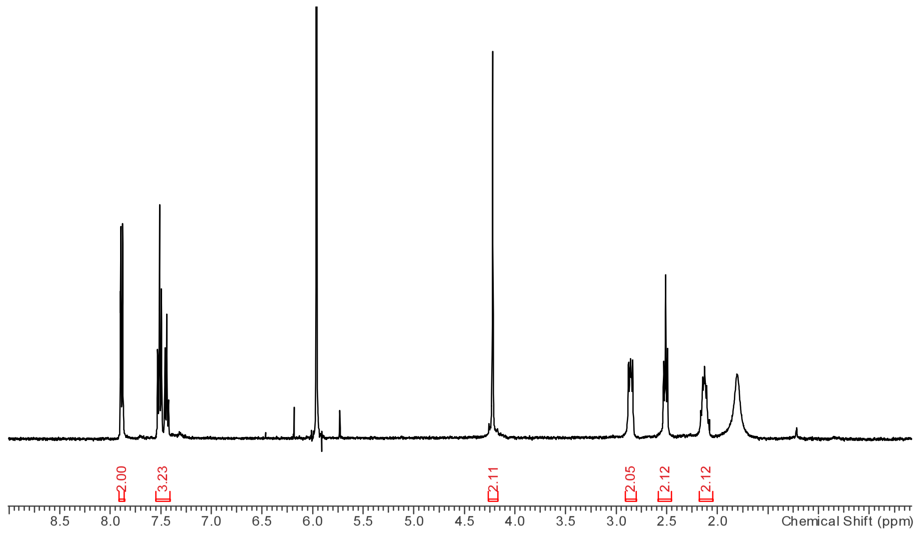

Figure S3. ^{1}H-NMR spectra of **(PCB)$_2$C$_2$** recorded at 293 K in 1,1,2,2-tetrachloroethane-d_2.

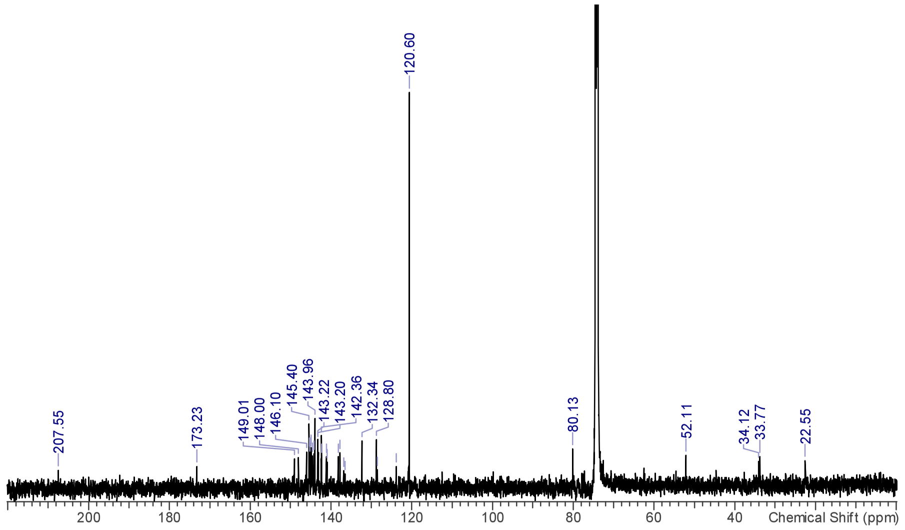

Figure S4. ^{13}C-NMR spectra of **(PCB)$_2$C$_2$** recorded at 293 K in 1,1,2,2-tetrachloroethane-d_2.

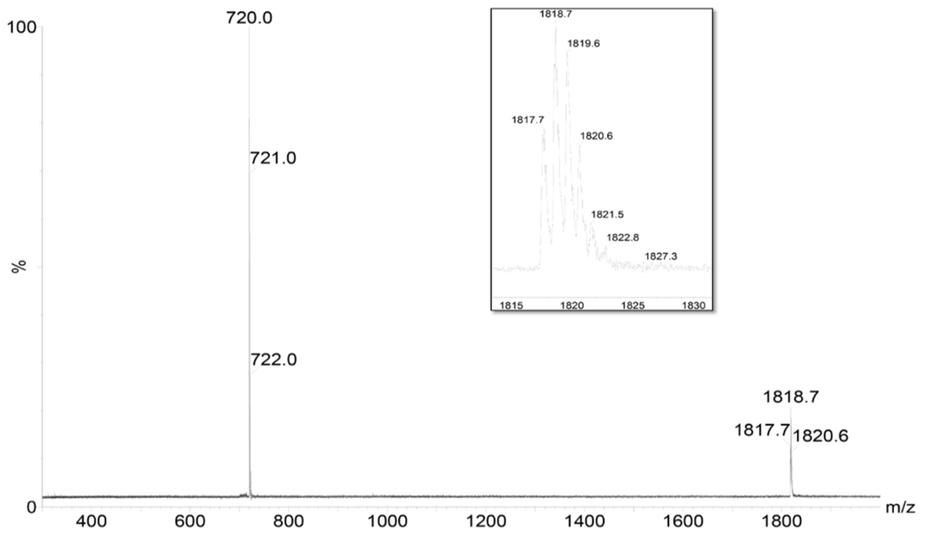

Figure S5. Negative ion MALDI mass spectrum of **$(PCB)_2C_2$**, using C_{60} (m/z: 720.0) as internal calibrant. The inset shows a zoomed view of the molecular ion peak.

[6,6]-Phenyl-C61-butyric acid 2-hydroxytetradeuteroethyl ester (*d*-$PCBC_2OH$). [6,6]-Phenyl-C61-butyric acid (**PCBA**) (400 mg, 0.45 mmol) was added to 80 mL of anhydrous *o*-dichlorobenzene and sonicated during 20 minutes. After sonication the brownish suspension was heated to 110°C for two hours in order to fully dissolve the PCBA. The resulting black solution was cooled to 0°C in an ice-bath before ethylene glycol-d_4 (1 mL, 7.85 mmol) and 4-Dimethylaminopyridine (5.5 mg, 0.05 mmol) were added. Finally *N,N'*-Dicyclohexylcarbodiimide (101 mg, 0.49 mmol) was added to the reaction mixture, which was allowed to warm to room temperature and stirred overnight. The solvent was removed from the reaction mixture by rotary evaporation. The recovered crude product was purified by column chromatography on silica gel using a mixture of toluene:pyridine (95:5) as mobile phase. The recovered fractions were concentrated and precipitated into cold methanol. The title compound was recovered as a brown solid after filtration (107 mg, 0.11 mmol, 25 %). ^{1}H NMR (400 MHz, 1,1,2,2-tetrachloroethane-d_2) δ ppm 7.97 - 7.88 (m, 2 H), 7.60 - 7.42 (m, 3 H), 2.96 - 2.80 (m, 2 H), 2.55 (t, *J*=7.6 Hz, 2 H), 2.21 - 2.06 (m, 2 H). MS (MALDI-ToF): *m/z* calcd for $C_{73}H_{12}D_4O_3$ ($M^{·-}$) 944.1, 945.1, 946.1, 947.1 found 944.1, 945.1, 946.1, 947.1.

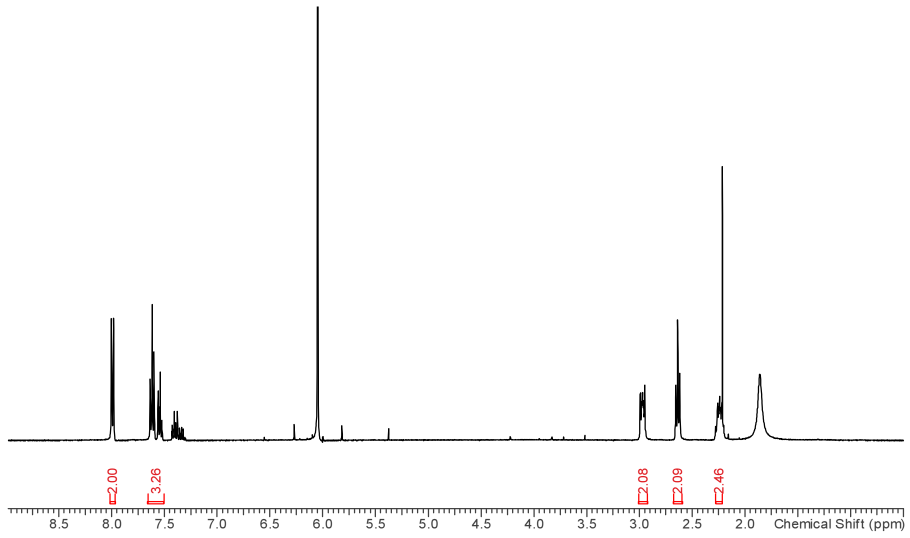

Figure S6. ^{1}H-NMR spectra of ***d*-PCBC$_2$OH** recorded at 293 K in 1,1,2,2-tetrachloroethane-*d*$_2$.

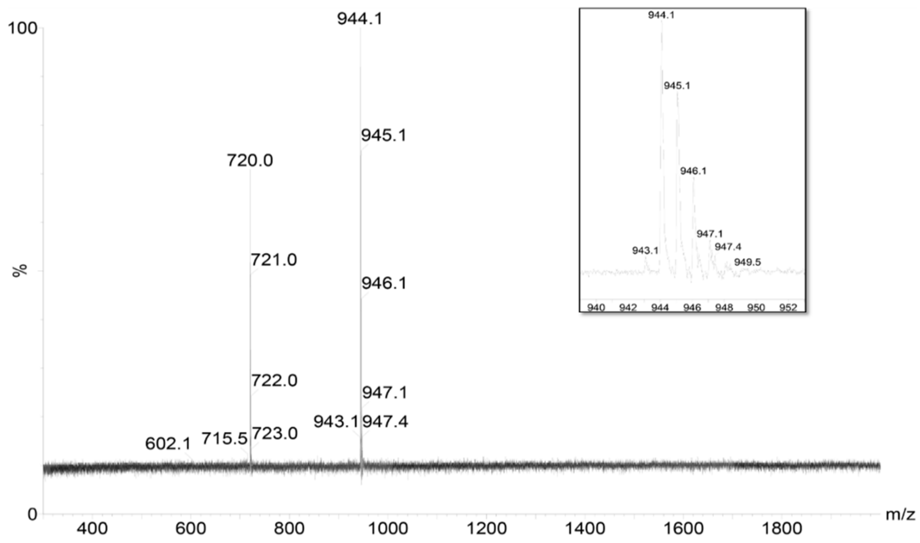

Figure S7. Negative ion MALDI mass spectrum of ***d*-PCBC$_2$OH**, using C_{60} (m/z: 720.0) as internal calibrant. The inset shows a zoomed view of the molecular ion peak.

Bis-[6,6]-phenyl-C61-butyric acid tetradeuteroethane-1,2-diyl ester (*d*-(PCB)$_2$C$_2$).

d-PCBC$_2$OH (95 mg, 0.10 mmol) was dissolved in 50 mL of anhydrous *o*-dichlorobenze and cooled to 0°C. PCBA (180 mg, 0.20 mmol) and 4-Dimethylaminopyridine (2.5 mg, 0.02 mmol) were added, before *N,N'*-Dicyclohexylcarbodiimide (45 mg, 0.22 mmol) was added. The reaction mixture was allowed to warm to room temperature and stirred overnight. Afterwards the solvent was removed from the reaction mixture and the recovered crude product was purified by column chromatography on silica gel using toluene as eluent. The recovered

fractions were concentrated and precipitated into methanol. The title compound was recovered as a dark brown solid after filtration (26 mg, 0.01 mmol, 14%). ^{1}H NMR (400 MHz, 1,1,2,2-tetrachloroethane-*d*$_2$) δ ppm 7.99 - 7.96 (m, 2 H), 7.63 - 7.50 (m, 3 H), 2.97 - 2.92 (m, 2 H), 2.60 (t, *J*=7.4 Hz, 2 H), 2.24 - 2.19 (m, 2 H). MS (MALDI-ToF): *m/z* calcd for $C_{144}H_{22}D_4O_4$ ($M^{\cdot -}$) 1823.2, 1824.2, 1822.2, 1825.2 found 1822.7, 1823.7, 1821.7, 1824.6.

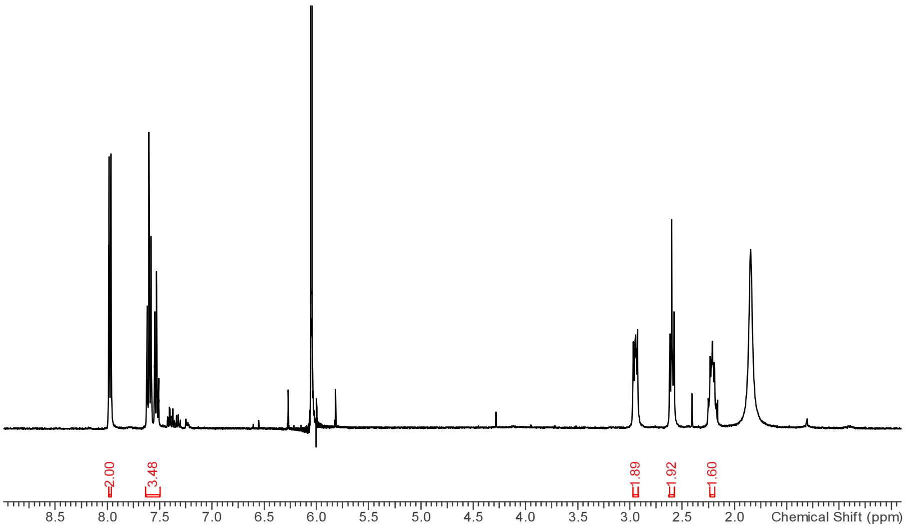

Figure S8. ^{1}H-NMR spectra of ***d*-(PCB)$_2$C$_2$** recorded at 293 K in 1,1,2,2-tetrachloroethane-*d*$_2$.

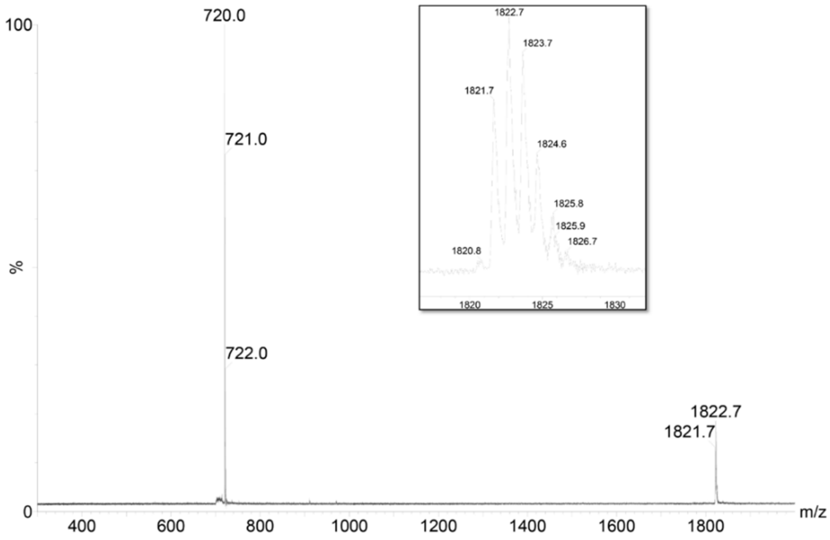

Figure S9. Negative ion MALDI mass spectrum of ***d*-(PCB)$_2$C$_2$**, using C_{60} (m/z: 720.0) as internal calibrant. The inset shows a zoomed view of the molecular ion peak.

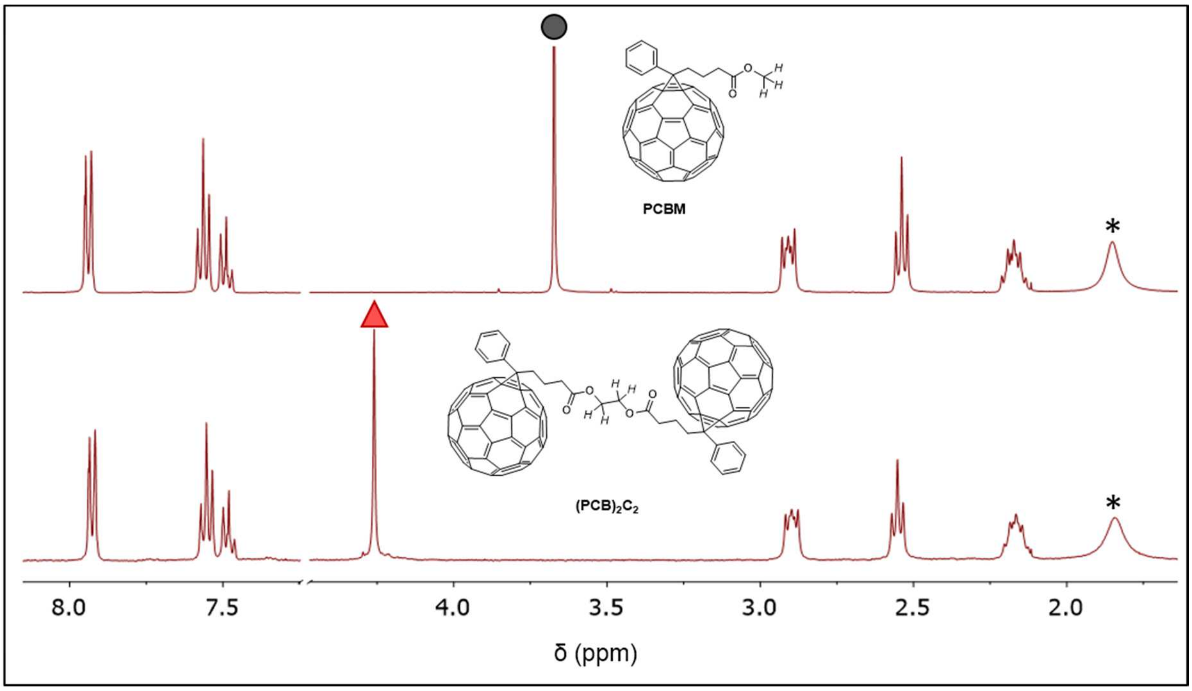

Figure S10. ^{1}H-NMR spectra of PCBM (top) and $(PCB)_2C_2$ (bottom) recorded at 293 K in 1,1,2,2-tetrachloroethane-d_2. In the PCBM spectrum, the singlet attributed to the terminal methyl protons is highlighted with a black circle. Similarly the singlet assigned to the bridging methylene protons in the $(PCB)_2C_2$ spectrum are marked by a red triangle. The broad peak at 1.84 ppm (marked by a *) originates from residual water in the NMR solvent.

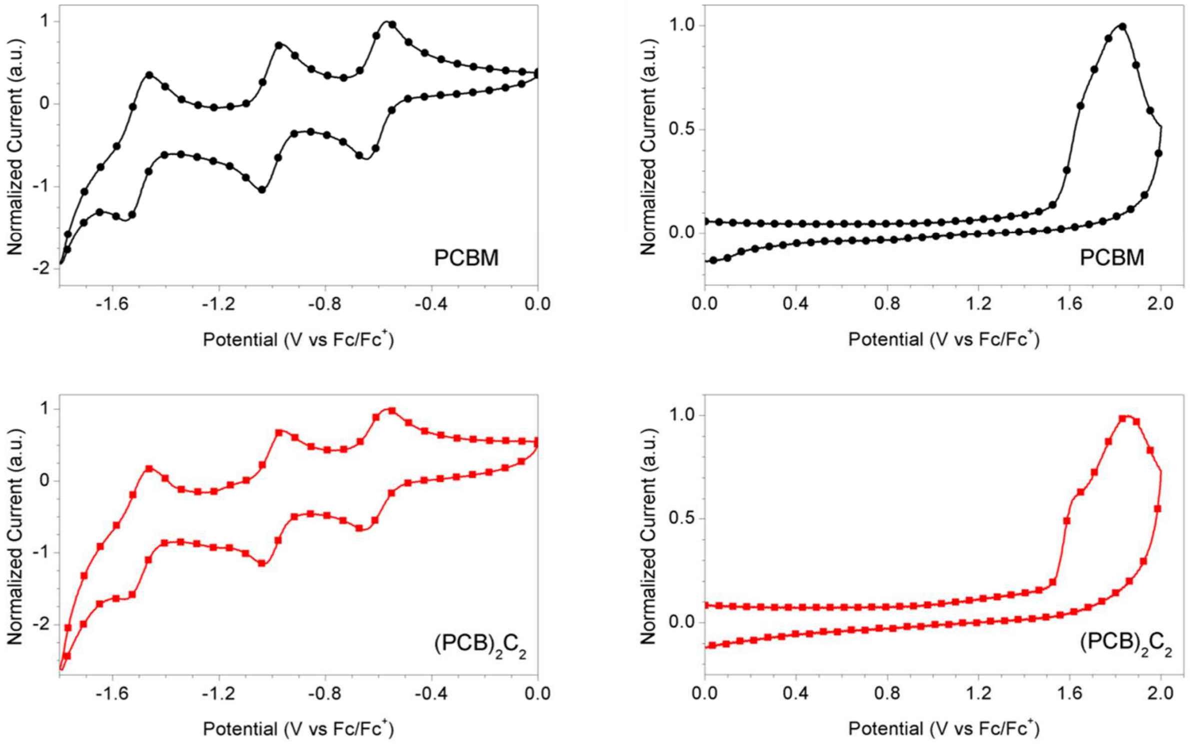

Figure S11. Cyclic voltammograms **PCBM** (top), respectively **(PCB)$_2$C$_2$** (bottom) were recorded at a scan rate of 100 mV/s in anhydrous *o*-dichlorobenzene solution with 0.1 M tetrabutylammonium hexafluorophosphate as the supporting electrolyte. Reductions are shown on the left and the oxidation cycles on the right.

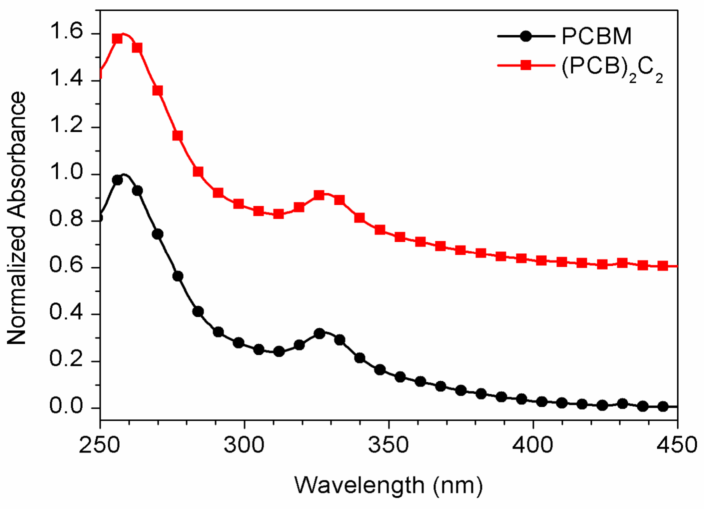

Figure S12. UV-*vis.* absorption spectra of **PCBM** and **(PCB)$_2$C$_2$** were recorded in dilute THF solutions at 25°C.

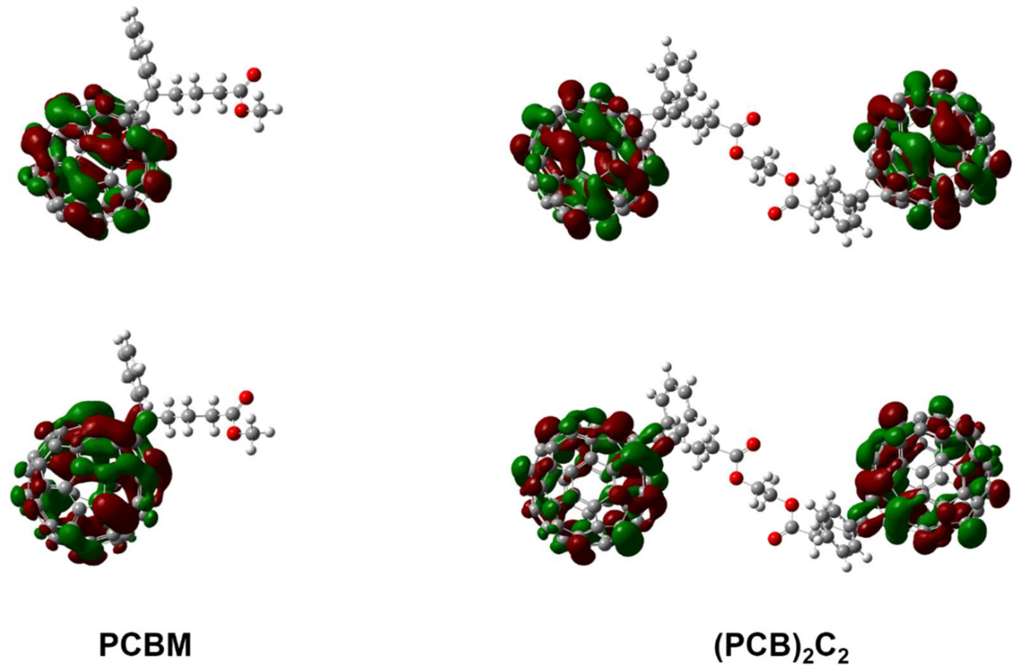

Figure S13. Energy-minimized structure (B3LYP/6-31G*) of **PCBM** and **(PCB)$_2$C$_2$** with visualization of the HOMO (bottom row) and LUMO (top row) wave functions. In case of

(PCB)$_2$C$_2$, both HOMO and LUMO were found to be degenerate and the above wave function distributions represent the sum of both degenerate states, HOMO+(HOMO-1) and LUMO+(LUMO+1).

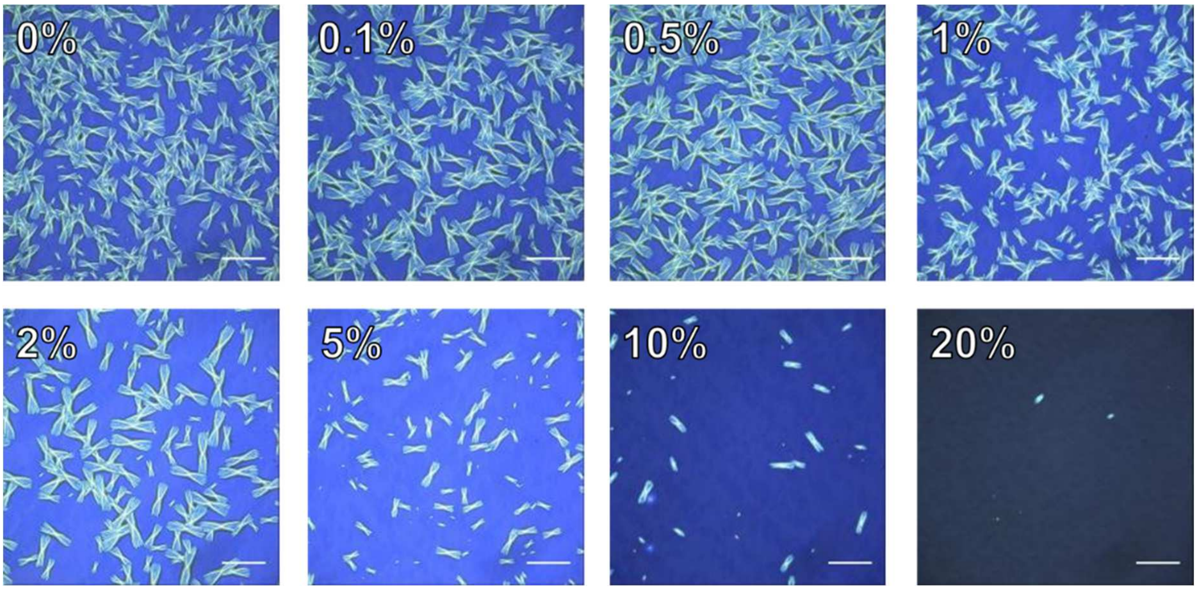

Figure S14. Optical microscope images of **PCDTBT**:fullerene (w/w 1:2) blends on silicon substrate with an increasing percentage of **(PCB)$_2$C$_2$**. All blend films were annealed under identical condition for 1 hour at 140°C prior to the measurement. Scale bar is 25 µm.

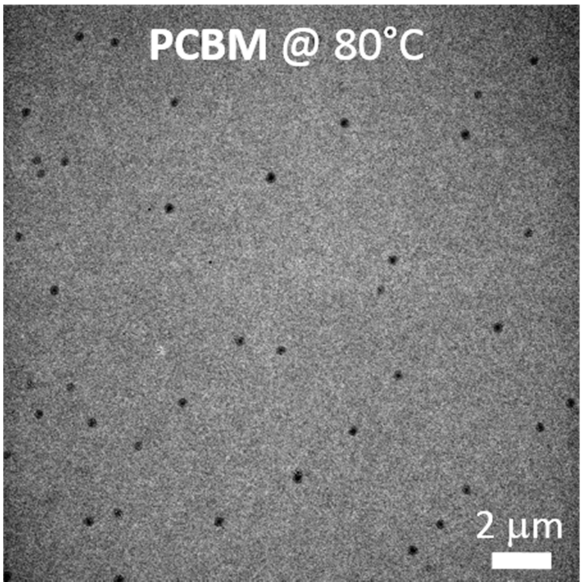

Figure S15. TEM micrograph of **PCDTBT:PCBM** film annealed 80°C prior to the measurement. Scale bar is 2 µm.

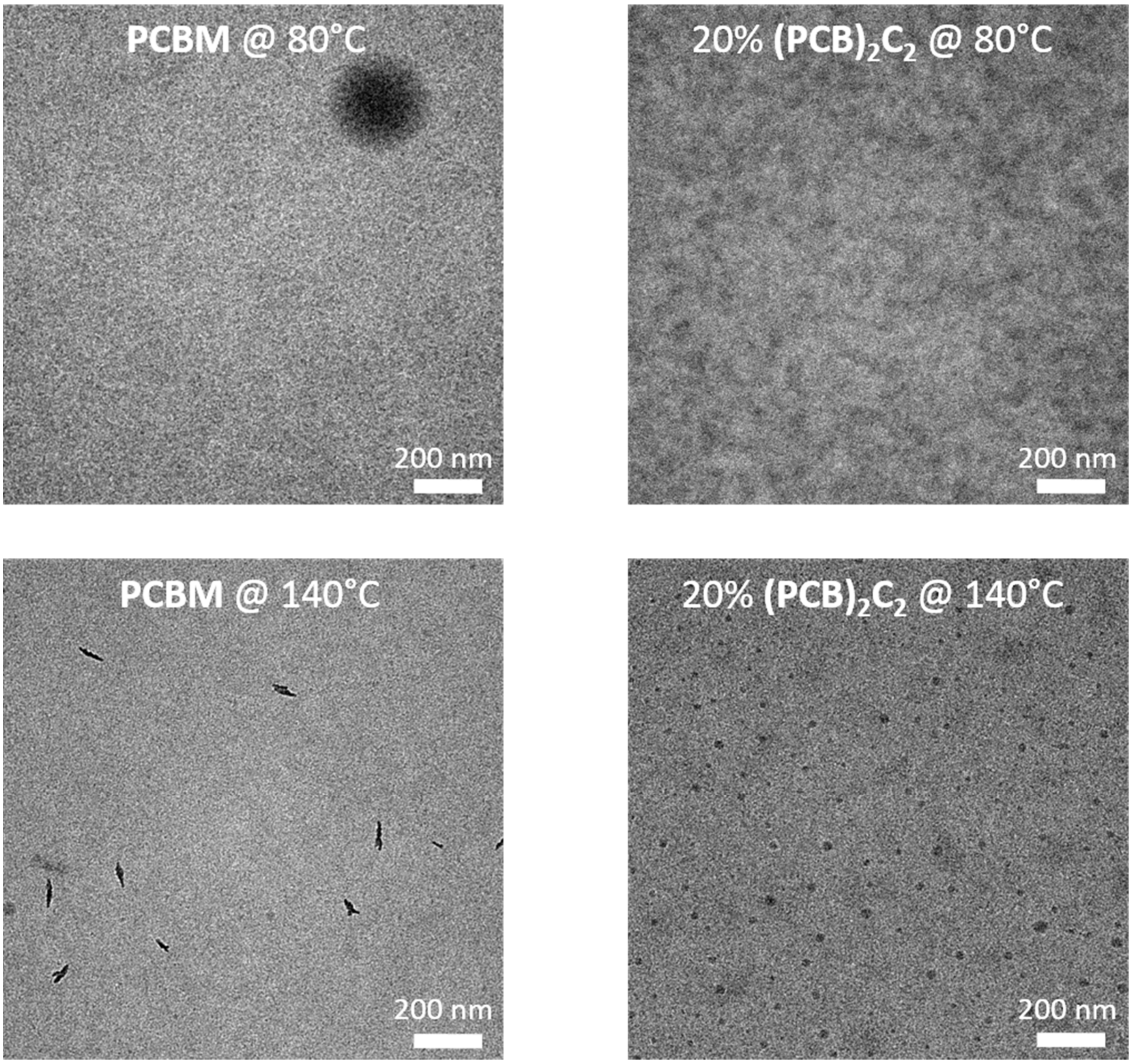

Figure S16. TEM micrographs of **PCDTBT:PCBM** film (left column) and **PCDTBT:PCBM:(PCB)$_2$C$_2$** (20%) (right column) annealed at the indicated temperatures prior to the measurements. Scale bar is 200 nm.

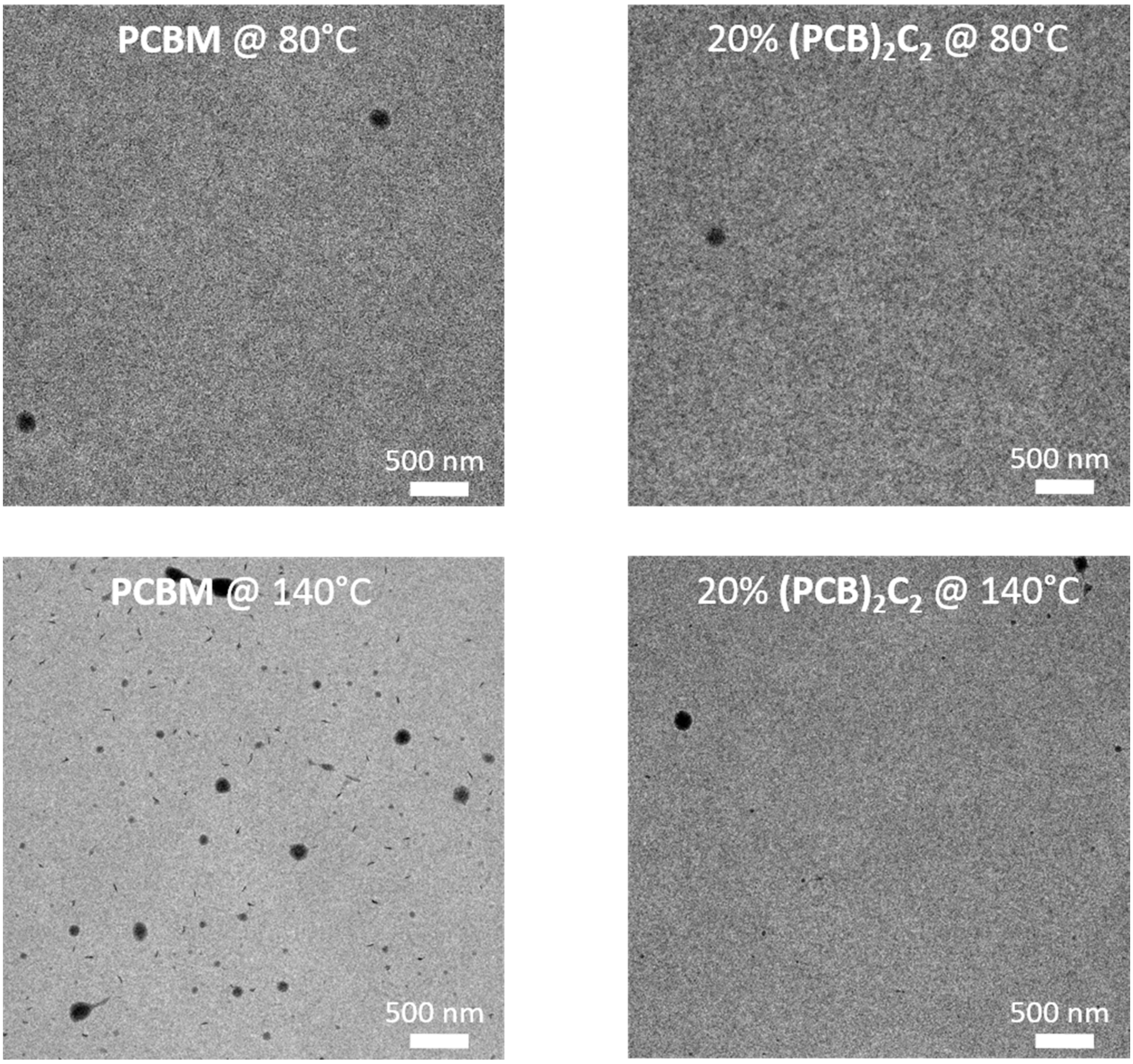

Figure S17. TEM micrographs of **PCDTBT:PCBM** film (left column) and **PCDTBT:PCBM:(PCB)$_2$C$_2$** (20%) (right column) annealed at the indicated temperatures prior to the measurements. Scale bar is 500 nm.

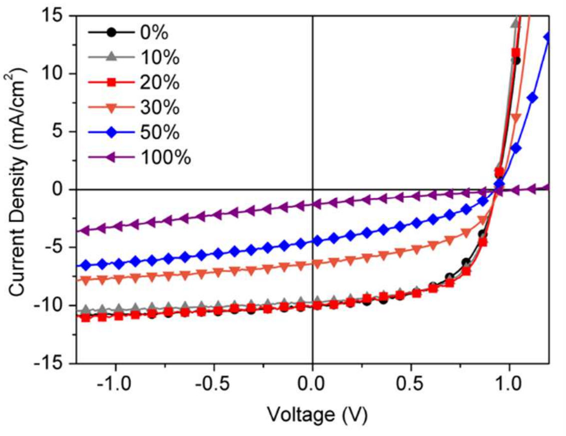

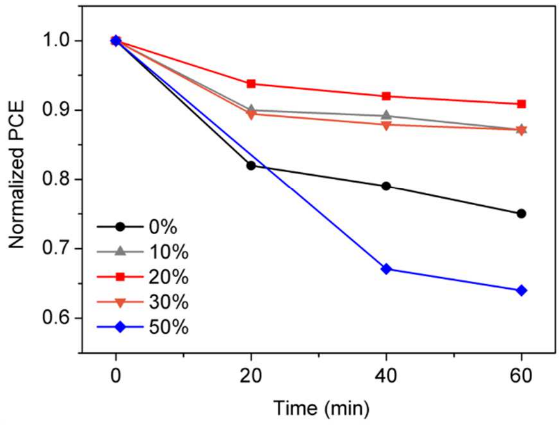

Figure S18. (Left) Comparison of the initial J-V characteristics of optimized conventional **PCDTBT:PCBM** devices containing different weight percentages of **$(PCB)_2C_2$** prior to thermal stability test. (Right) Degradation of solar cell PCE as a function of time at 85°C thermal stress in nitrogen atmosphere of optimized conventional **PCDTBT:PCBM** devices containing different weight percentages of **$(PCB)_2C_2$**.

Table S1. Electrochemical, theoretical and optical properties of **PCBM** and **$(PCB)_2C_2$**.

	CV	DFT	UV-vis.
Compound	HOMO/LUMO [eV] [a]	HOMO/LUMO [eV] [b]	λ_{max} soln. [nm] [c]
PCBM	-5.94 / -3.85	-5.70 / -3.13	258, 328
$(PCB)_2C_2$	-5.94 / -3.85	-5.71 / -3.14	258, 328

[a] Measured in *o*-dichlorobenzene solution with 0.1 M $TBAPF_6$ as supporting electrolyte. Scan rate 100 mV/s. [b] Energy levels calculated by DFT using the B3LYP/6-31g* model. [c] Recorded in dilute THF solution at 25°C.

Table S2. Initial device parameters of conventional **PCDTBT:PCBM** devices containing different loadings of the **(PCB)₂C₂**.

System	J_{sc} [mA/cm^2]	V_{oc} [V]	FF	PCE [%]
PCDTBT:PCBM	10.06	0.93	0.56	5.24
PCDTBT:PCBM:$(PCB)_2C_2$ (10%)	9.70	0.93	0.61	5.50
PCDTBT:PCBM:$(PCB)_2C_2$ (20%)	10.01	0.93	0.62	5.77
PCDTBT:PCBM:$(PCB)_2C_2$ (30%)	6.40	0.95	0.50	3.04
PCDTBT:PCBM:$(PCB)_2C_2$ (50%)	4.54	0.93	0.38	1.60
PCDTBT:$(PCB)_2C_2$ (100%)	1.34	1.07	0.21	0.30

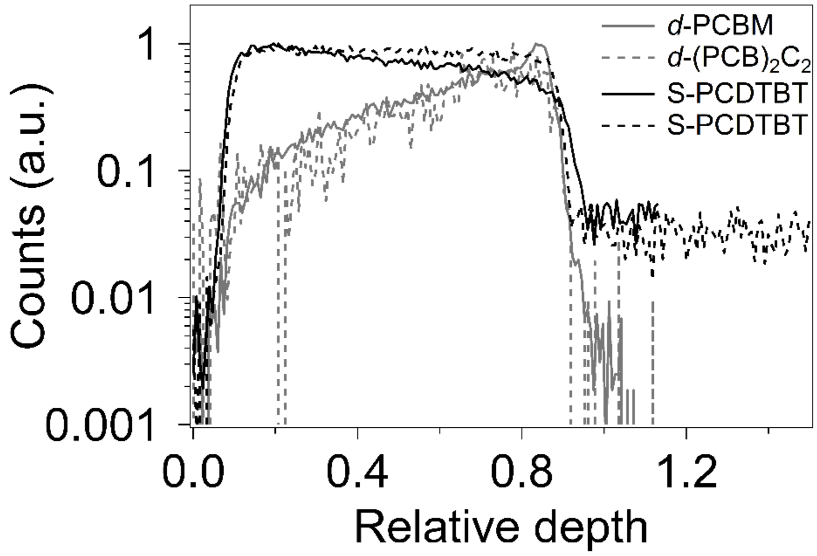

Figure S19. DSIMS profiles of ^{2}H and ^{32}S in a **PCDTBT:*d*-PCBM** bilayer (solid lines) and in a **PCDTBT:(BHJ PCDTBT:*d*-(PCB)₂C₂)** bilayer (dotted line) fabricated on a SiO_2/Si substrate. Partial infiltration of the deuterated fullerene derivatives into the **PCDTBT** layers is observed at annealing temperatures of 100°C.

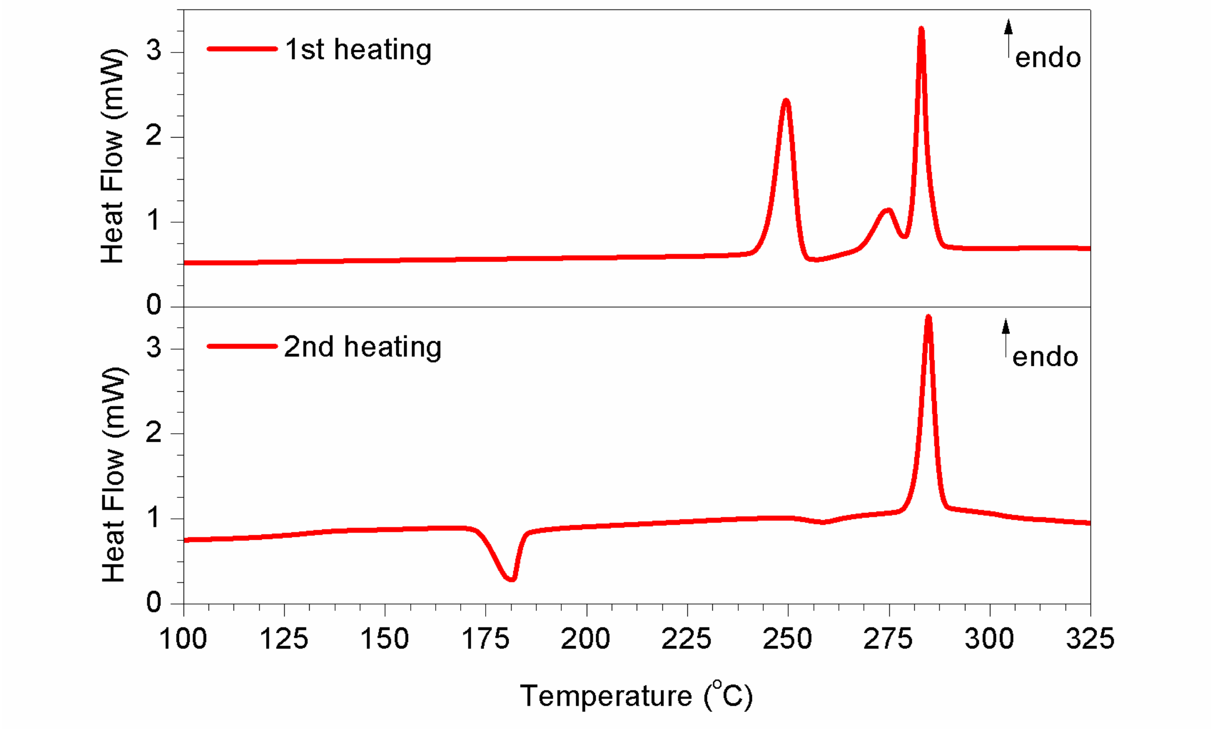

Figure S20. DSC traces of **PCBM** recorded at heating rate of 10°C/min.

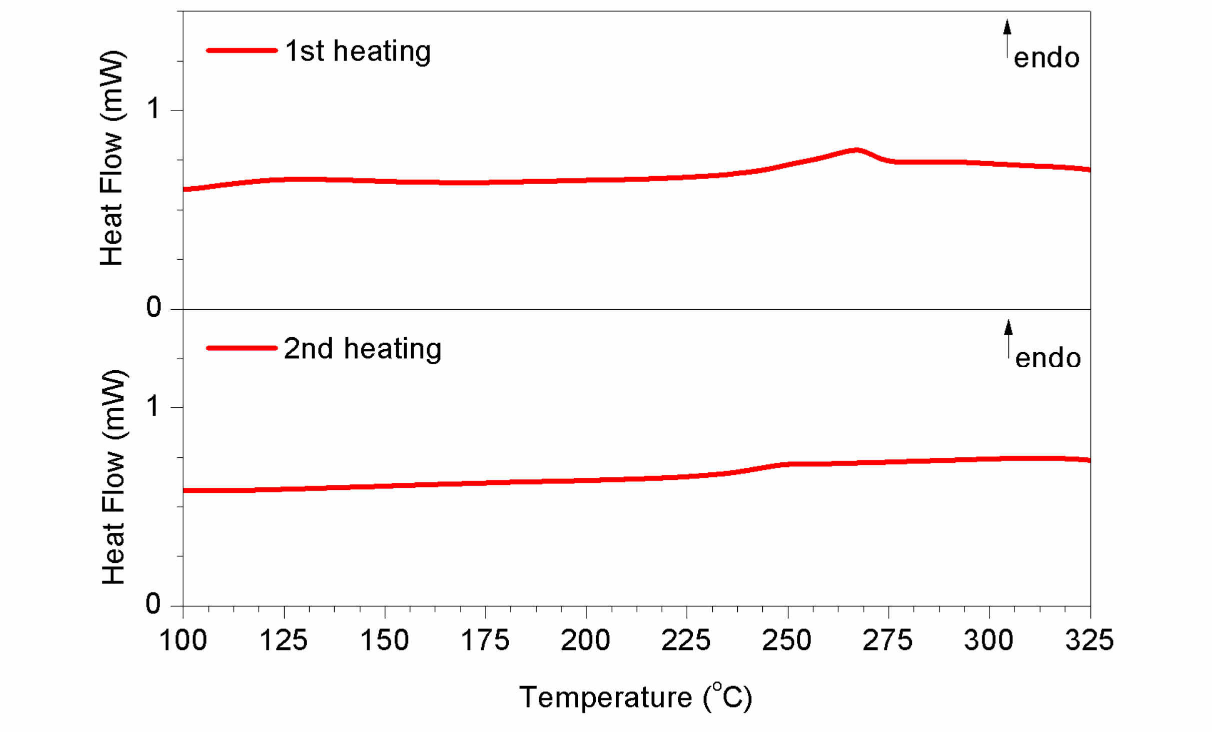

Figure S21. DSC traces of **$(PCB)_2C_2$** recorded at heating rate of 10°C/min..

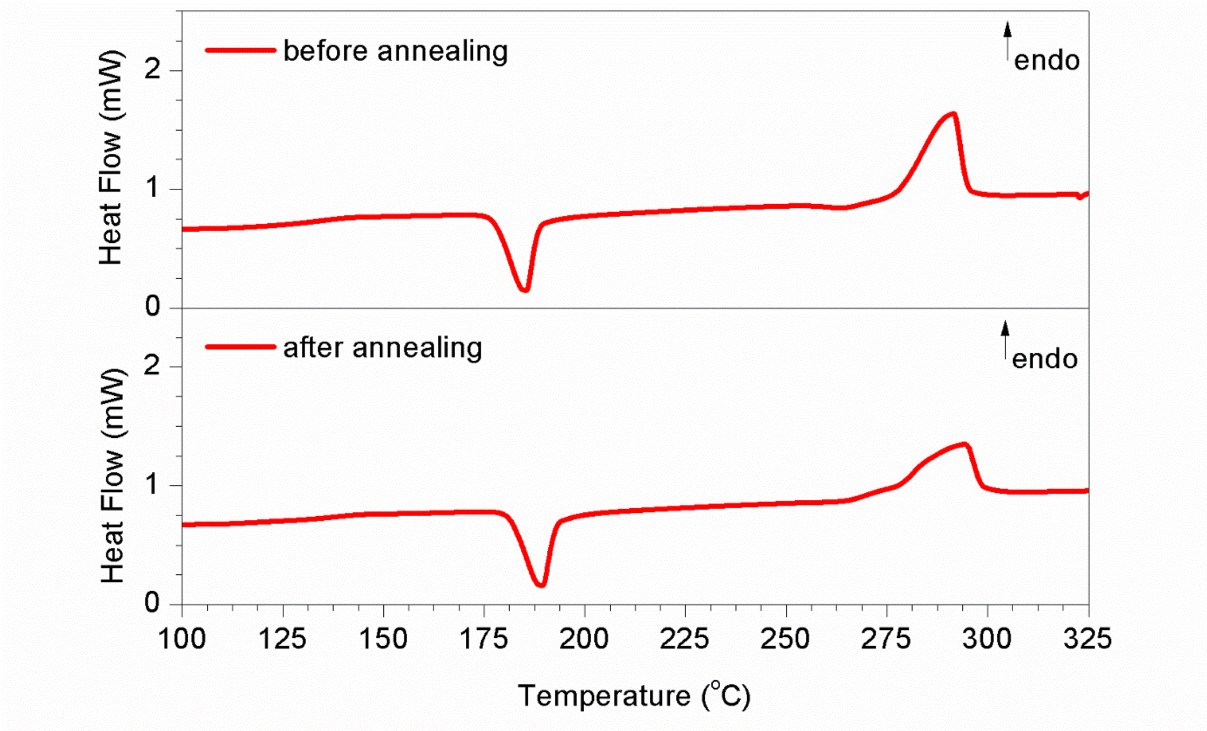

Figure S22. DSC traces of **PCBM** before 2 hour annealing at 85°C and after annealing, recorded at heating rate of 10°C/min..

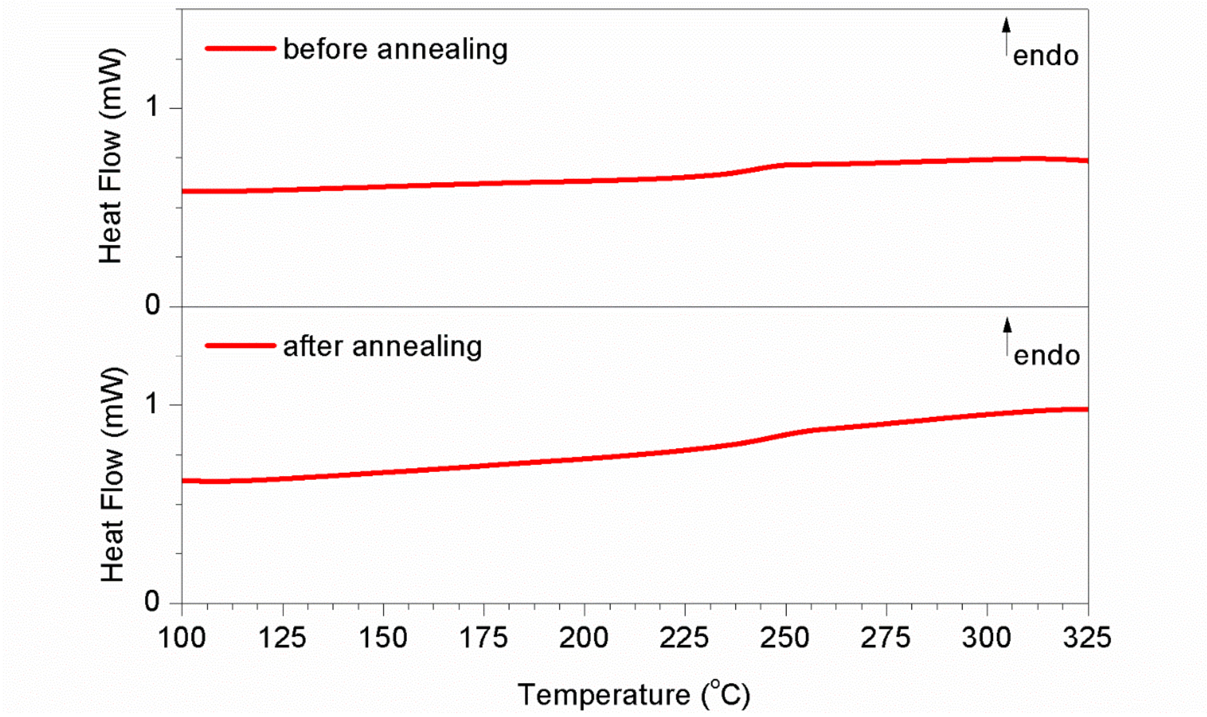

Figure S23. DSC traces of **$(PCB)_2C_2$** before 2 hour annealing at 85°C and after annealing, recorded at heating rate of 10°C/min..

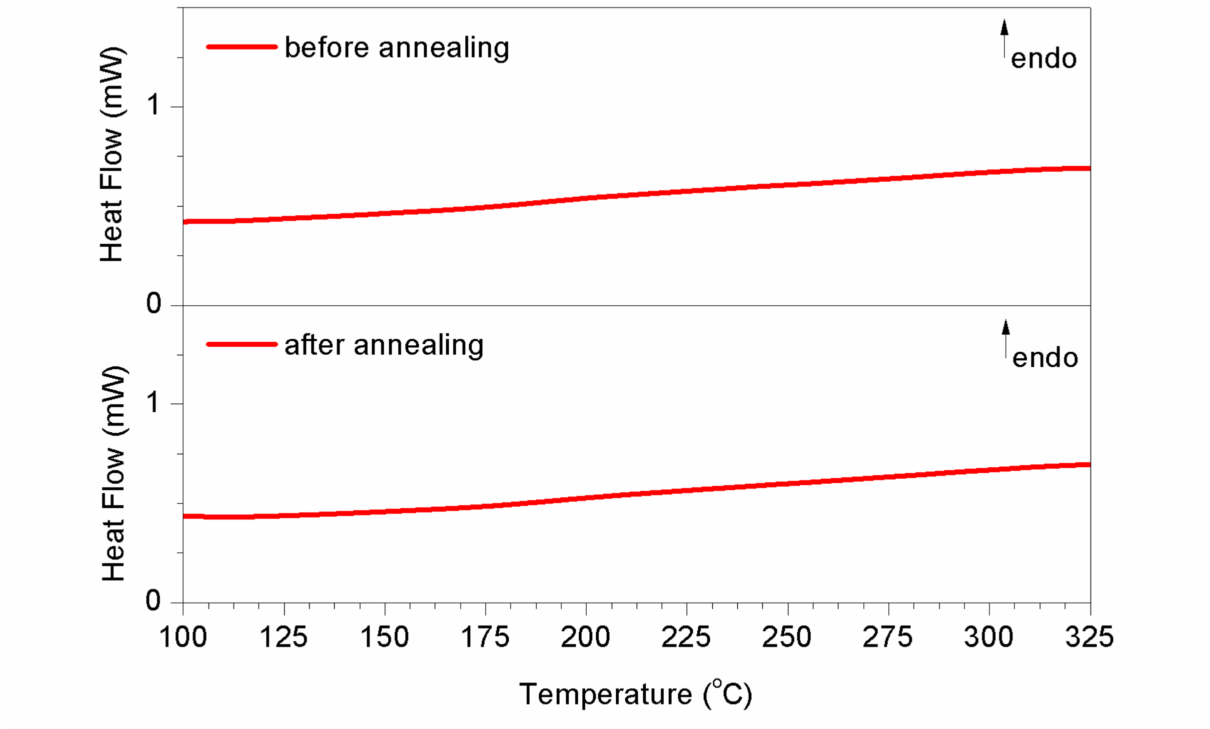

Figure S24. DSC traces of **PCBM:$(PCB)_2C_2$** (20%) before 2 hour annealing at 85°C and after annealing, recorded at heating rate of 10°C/min.

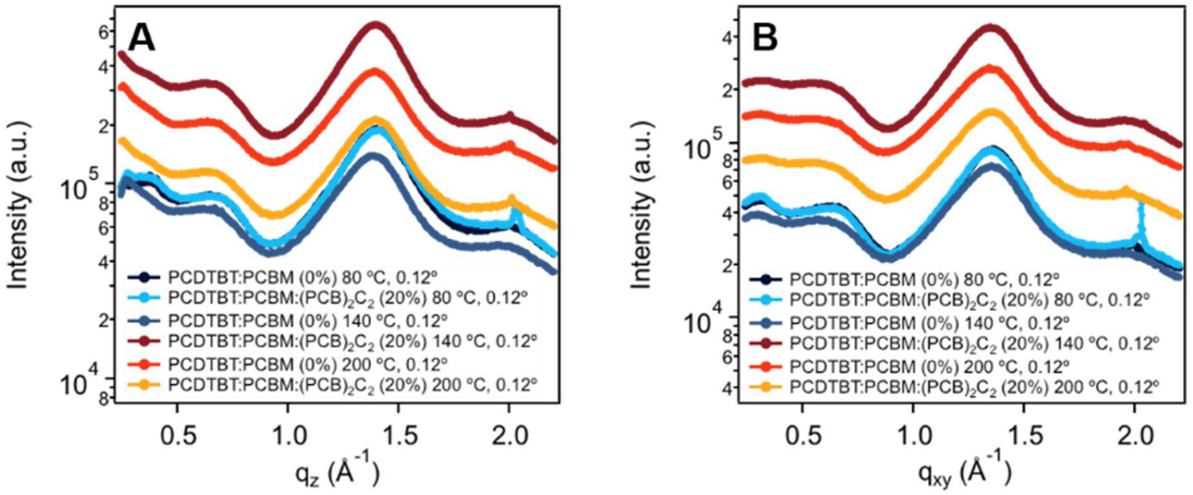

Figure S25. (A) out-of-plane and (B) in-plane GIWAXS diffraction patterns of **PCDTBT:PCBM** and **PCDTBT:PCBM:$(PCB)_2C_2$** (20%) BHJ films, annealed at various temperatures prior to the measurements.

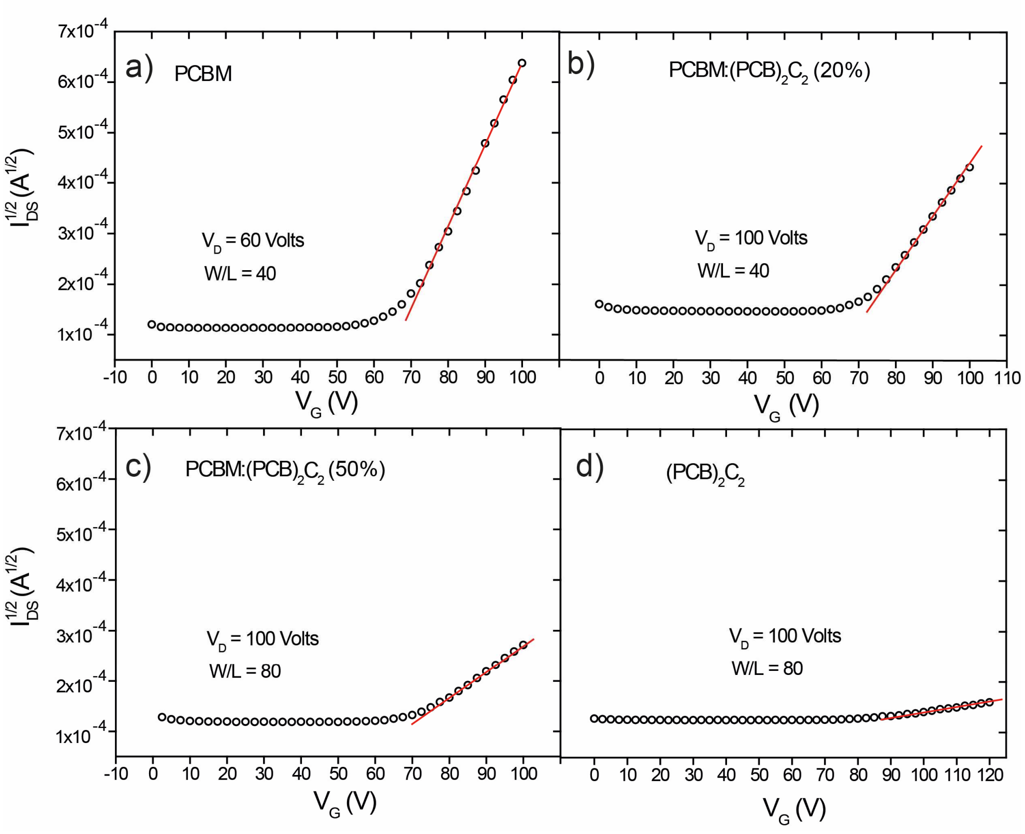

Figure S24. Transfer curves of **PCBM:(PCB)$_2$C$_2$** films for different weight ratios. As shown, the gate-induced current decreases as the weight percent of the dimer increases.

[1] Q. Wei, T. Nishizawa, K. Tajima, K. Hashimoto, *Advanced Materials* **2008**, *20*, 2211-2216.
